# Designing a Distributed Ledger Technology System for Interoperable and General Data Protection Regulation–Compliant Health Data Exchange: A Use Case in Blood Glucose Data

**DOI:** 10.2196/13665

**Published:** 2019-06-14

**Authors:** David Hawig, Chao Zhou, Sebastian Fuhrhop, Andre S Fialho, Navin Ramachandran

**Affiliations:** 1 Pact Care BV Amsterdam Netherlands; 2 Centre for Health Informatics & Multiprofessional Education University College London London United Kingdom

**Keywords:** distributed ledger technology, directed acyclic graph, IOTA, IPFS, blockchain, Masked Authenticated Messaging, MAM, mobile health, blood glucose, diabetes, FHIR

## Abstract

**Background:**

Distributed ledger technology (DLT) holds great potential to improve health information exchange. However, the immutable and transparent character of this technology may conflict with data privacy regulations and data processing best practices.

**Objective:**

The aim of this paper is to develop a proof-of-concept system for immutable, interoperable, and General Data Protection Regulation (GDPR)–compliant exchange of blood glucose data.

**Methods:**

Given that there is no ideal design for a DLT-based patient-provider data exchange solution, we proposed two different variations for our proof-of-concept system. One design was based purely on the public IOTA distributed ledger (a directed acyclic graph-based DLT) and the second used the same public IOTA ledger in combination with a private InterPlanetary File System (IPFS) cluster. Both designs were assessed according to (1) data reversal risk, (2) data linkability risks, (3) processing time, (4) file size compatibility, and (5) overall system complexity.

**Results:**

The public IOTA design slightly increased the risk of personal data linkability, had an overall low processing time (requiring mean 6.1, SD 1.9 seconds to upload one blood glucose data sample into the DLT), and was relatively simple to implement. The combination of the public IOTA with a private IPFS cluster minimized both reversal and linkability risks, allowed for the exchange of large files (3 months of blood glucose data were uploaded into the DLT in mean 38.1, SD 13.4 seconds), but involved a relatively higher setup complexity.

**Conclusions:**

For the specific use case of blood glucose explored in this study, both designs presented a suitable performance in enabling the interoperable exchange of data between patients and providers. Additionally, both systems were designed considering the latest guidelines on personal data processing, thereby maximizing the alignment with recent GDPR requirements. For future works, these results suggest that the conflict between DLT and data privacy regulations can be addressed if careful considerations are made regarding the use case and the design of the data exchange system.

## Introduction

The delivery of high-quality health care requires the efficient and effective exchange of patient data [1,2]. To that end, health care systems across the globe have invested heavily over the past decade in a variety of solutions and tools that aim to improve the interoperable exchange of data, including health information exchanges, direct messaging, community clouds, and open application programming interfaces (APIs) [3]. These solutions have achieved different degrees of success in the real world, largely due to common concerns regarding privacy and security [4].

Distributed ledger technology (DLT) is a new type of database technology in which databases are connected in a distributed fashion through a peer-to-peer network and maintained by a consensus protocol [5]. Proponents of DLT believe that it offers great potential to make the exchange of health information immutable and secure [6-8]. However, the immutable and transparent character of these technologies may conflict with data privacy regulations or data processing best practices.

The European Union has recently instituted the General Data Protection Regulation (GDPR), which regulates the collection, processing, and securing of personal data, including protected health information (PHI). This new regulation sets out the way by which personal data are to be protected as well as defining the main rights of the person to whom the data relates (ie, the data subject): right of rectification, right to erasure, right of access, and rights related to automated processing. As detailed by the European Union Blockchain Observatory and Forum [9], in principle, there are no contradictions between the goals of GDPR and DLT. However, there are three areas in which GDPR still does not offer enough clarity about how real-world DLT applications should be developed. These areas include (1) accountability and roles (eg, how to identify a data controller in a public DLT), (2) anonymization of personal data (eg, what techniques are sufficient to anonymize personal data to the point where the resulting output can potentially be stored in a DLT), and (3) GDPR rights conflicts (eg, how to rectify or remove personal data that are recorded in a DLT that is immutable by nature, or who is responsible for requesting and managing the “freely, specific, informed, and unambiguous” consent from a data subject, especially if the data controller is not specified) [10].

With regards to anonymization of personal data, it is clear that GDPR does not apply to anonymized data and that this type of information can be stored on the DLT. However, what qualifies as anonymized is still not clear. The only indication today is that it must be irreversibly impossible to identify an individual through any of the means “reasonably likely to be used” [11]. Within the health care context, achieving this irreversible identification is even more difficult as PHI includes not only general personal data but also health status information, genetic data, and biometric data. In this way, GDPR sets a new bar for health care data anonymization compared to the “pseudonymization” methods currently used in clinical research, and where confidentiality is ensured through a simpler key coding of the data.

At this stage, the guidance provided by GDPR on how to process personal data simply refers to the need to minimize both the risk of reversal (eg, risk of reversing and reconstituting the original data) and the risk of linkability (eg, risk of linking anonymized data to an individual by examining patterns of usage or context, or by comparison to other pieces of information) [12,13]. A variety of techniques may accomplish this data anonymization, including obfuscation, encryption, hashing, and aggregation [14]. However, it remains unclear from a legal perspective, which of them (individually or in combination) are most adequate to convert personal data into anonymous data [9].

**Figure 1:**
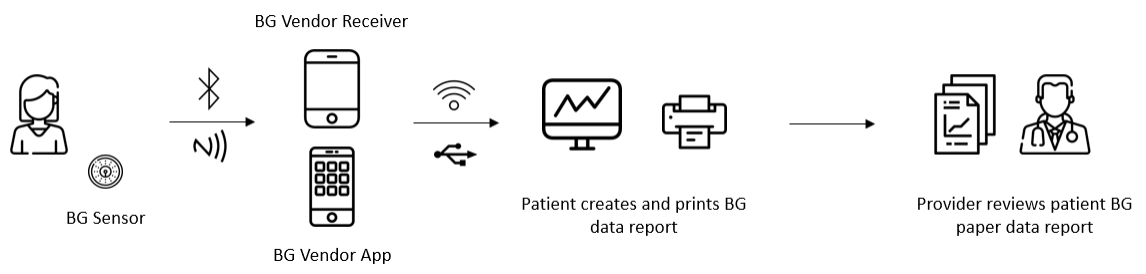
Typical steps for current patient-provider exchange of continuous blood glucose (BG) data: (1) an adhesive patch holding the BG sensor is attached to the patient’s skin and measures glucose readings in interstitial fluid throughout the day and night; (2) the sensor sends real-time readings wirelessly to a receiver/smart device app, so the user can view the information; (3) the receiver or smart device app displays current and historical glucose levels and allows for this data to be printed and/or exported (eg, .txt file); and (4) the patient and provider review together the paper notes or exported files.

In this way, given the importance of minimizing the risk of data reversal and linkability for compliance with existing data privacy regulations, this study explores the potential of one specific DLT in supporting health information exchange by developing a proof-of-concept system for immutable, automated, and secure exchange of patient health information. Specifically, we focused on the patient-provider exchange of blood glucose (BG) data, not only because of the large number of patients affected by diabetes worldwide but also due to the importance of patient-provider communication of this data for improved treatment management. At present, patients usually track their glucose levels manually or rely on the vendor’s software to compile a report (Figure 1). This manual process is time-consuming and error-prone [15], highlighting the need for systems supporting data exchange between self-monitoring BG devices and electronic health records (EHRs) in a secure, effective, and tamper-proof way.

## Methods

### System Design

**Figure 2:**
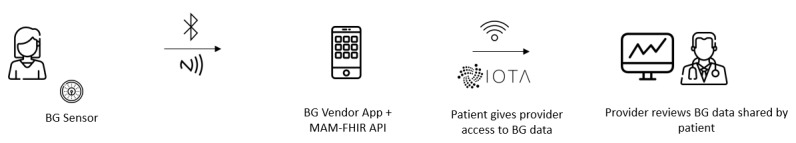
Proposed steps for a distributed ledger technology (DLT)-based patient-provider exchange of blood glucose (BG) data: (1) an adhesive patch holding the BG sensor is attached to the patient’s skin and measures glucose readings in interstitial fluid throughout the day and night; (2) the sensor sends real-time readings wirelessly to a smart device app, so the user can view the information; (3) the smart device app displays current and historical glucose levels and is connected to an application programming interface (API; “MAM-FHIR API”) that allows for these data to be exported to a DLT; and (4) if a patient provides consent, the interoperable data stored on the DLT can be automatically exported to a physician’s electronic health record so that they can be reviewed.

We aimed to develop a proof-of-concept system for patient-provider exchange of glucose data (Figure 2) according to the following specifications: (1) patient-controlled, (2) fully digital, (3) interoperable, and (4) distributed logging and storage of data. *Patient-controlled* means that patients, in addition to providing consent for exchange, can also grant data access to selected parties. *Digital* specifies that data are exchanged in a digital format (from the BG remote sensor acquisition to the upload into a physician’s EHR), minimizing any potential manual data entry errors. *System interoperability* specifies that the data generated by the BG remote sensor can be read, if consent is provided by the patient, by another information system from different health care parties (leveraging the Fast Healthcare Interoperability Resources [FHIR] standard). *Distributed logging and storage of data* means that all the data generated by the BG sensor are stored in a tamper-proof way across multiple distributed nodes instead of a single, centralized data repository.

Our system is built on four main modules: (1) a data conversion module which converts raw BG data from the device sensor into FHIR standard records, (2) a data processing module that transforms the generated FHIR records into a format that minimizes the risk of reversal and linkability, (3) a data storage and logging module which uploads the transformed FHIR records on a DLT, and (4) a key exchange module that allows establishing a patient-provider communication channel for the FHIR records to be exchanged.

The actual integration of the data from the DLT into a physician’s EHR is out of the scope of this work because it is dependent on the EHR system and integration engine. However, the use of the FHIR standard in the proposed designs should make this a relatively straightforward process. Additionally, we assume the use case in which physicians will pull the data from the system on a periodic quarterly basis and not on a continuous, real-time basis. This sampling choice is based on the current patient-provider workflows and guidelines across several health care systems (eg, in Germany, diabetic patients have quarterly visits with physicians to review and update the disease management plan).

Also out of the scope of this work is the issue of data cache when patients are offline or in cases of network connections issues. In general, there are two points where data can be cached: (1) at the BG sensor and/or (2) on the mobile device used to read the data from the sensor. However, the exact architecture of the data cache implementation is highly dependent on the sensor and device manufacturer. Usually, these manufacturers leverage different libraries that provide access to the different secure storage solutions (eg, for Samsung Galaxy devices it would be the ARM’s TrustZone).

**Figure 3:**
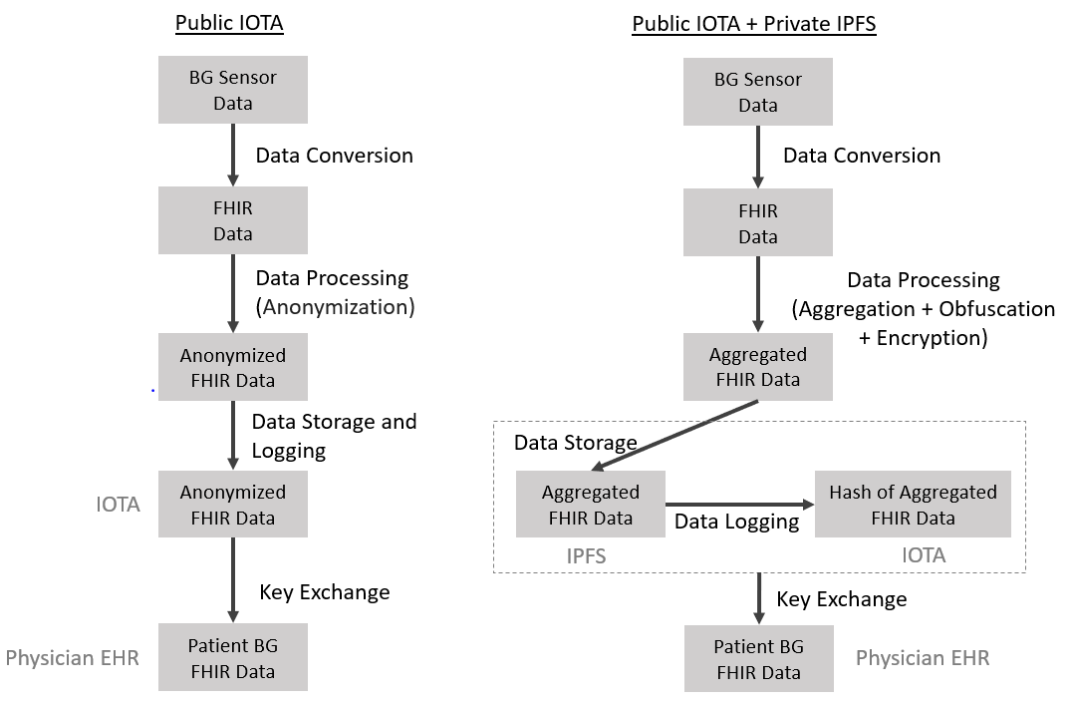
Proof-of-concept system for patient-provider exchange of blood glucose (BG) data with two variations: (1) public IOTA and (2) public IOTA plus private IPFS (InterPlanetary File System). EHR: electronic health record; FHIR: Fast Healthcare Interoperability Resources.

Given the ongoing debates around what techniques are suitable for PHI anonymization, and because there currently is not a standard design for patient-provider data exchange, we propose two different variations for our proof-of-concept system. Both variations aim at being compliant with GDPR and attempt to minimize the risk of reversal and linkability in their own way, with their own advantages and disadvantages. These two variations consist of the same four modules but differ in the sense that one is solely based on a public distributed ledger named IOTA, which we call “public IOTA,” and the second combines the IOTA public distributed ledger with an additional private distributed file system called InterPlanetary File System (IPFS), which we call “public IOTA plus private IPFS.” A summary of these two variations can be seen in Figure 3, and a more detailed description of each module is provided subsequently.

### Data Conversion Module

This module, common to both designs, converts raw data from the continuous BG system to the FHIR standard. FHIR was created by HL7 with the purpose of facilitating the interoperable exchange of health care-related data between different health care systems to make it easy to provide health care information to providers and individuals on a wide variety of devices and to allow third-party developers to provide medical apps that can be easily integrated into existing systems [16-18].

The FHIR-based records are built from a set of modular components called “Resources” that have standard, agreed-on data elements with consistent meaning across sharing entities. All resources share a common set of features including (1) resource identity and metadata, (2) a human-readable XHTML summary, (3) a URL that identifies the resource, and (4) a set of defined data elements (a different set for each type of resource).

For ease of use and standardization purposes, we used a GATT-compliant Bluetooth continuous glucose monitoring system (Dexcom G4 PLATINUM) [19]. This device generates one BG measurement every 10 minutes. Converting the data from this continuous glucose monitoring system to an FHIR record was done by filling an FHIR observation blueprint for each generated glucose data measurement (mmol/L). The output of this module is one FHIR JavaScript Object Notation (JSON) file per each BG measurement.

### Data Processing Module

#### Public IOTA

An FHIR record contains fields where PHI, such as patient name (eg, “John Doe”), date of birth (eg, “24-Sept 1932″), or medical record number (eg, ”123456″) may be present. For purposes of GDPR adherence in this system design, because FHIR records will be stored on the public IOTA DLT, data anonymization was performed. Through this module, we have simply removed any existent PHI entries from each FHIR JSON file. For naming purposes, we will refer to this processed data as *anonymized FHIR records.*

#### Public IOTA Plus Private IPFS

With this design, all FHIR records were stored on a private distributed file system (IPFS), and the resulting hash was logged into the public IOTA DLT. For purposes of GDPR compliance and to minimize the risk of data reversal and linkability, we applied a sequential combination of steps in this module: (1) data aggregation that combines multiple FHIR JSON records into one single JSON file, (2) data obfuscation based on the JavaScript Obfuscator using the proposed medium settings that are applicable for a JSON file [20], and (3) data encryption of the obfuscated JSON file using the AES256-GCM function with a tag length of 128, which is also recommended by the German Medical Association and adopted by the US government [21].

The sampling used in the aggregation step was based on the periodic quarterly visits assumed previously for the patient-provider interaction. This aggregation window is, however, a variable that can be easily set for different time intervals (eg, real time, daily, weekly). For naming purposes, we will refer to this processed data as *aggregated FHIR records.*

### Data Storage and Logging Module

#### Public IOTA

**Figure 4:**
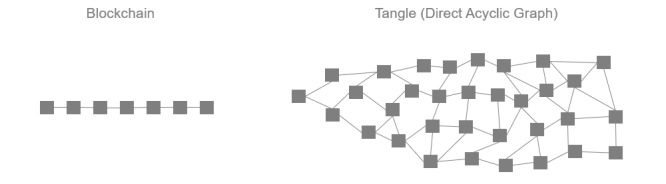
Sequential block-based transactions of a blockchain (left) and IOTA directed acyclic graph-based transactions (right).

As shown in Figure 3, this design variation was based on a public DLT protocol only. We selected IOTA’s DLT because it offers zero transaction fees and handles large transaction throughputs well. These properties make this technology particularly well-suited for data exchange across health care devices and systems [22]. What differentiates IOTA from blockchain-based DLTs is that transactions, instead of being grouped into blocks and stored in sequential chains, are linked together in a so-called Tangle (Figure 4). This design allows for a validation process that takes form as a Web structure referred to as a directed acyclic graph rather than a linked list as is the case in blockchain-based DLTs. Transactions on the Tangle, therefore, can get issued simultaneously, synchronously, and continuously. To issue a transaction on the Tangle, one must validate two other transactions. Therefore, the more participants use the system, the more transactions are validated, making it highly scalable. This “pay-it-forward” validation process also obviates the need for financial rewards to incentivize participants.

To share, store, and retrieve encrypted data, we used IOTA’s Masked Authenticated Messaging (MAM) module. This module encrypts messages (masking), confirms source origin (authentication), and creates a continuous message stream on the Tangle until the source stops publishing it (messaging). In a MAM stream, each message holds (1) the data, (2) a reference to the address of the next message only flowing in one direction (forward), and (3) a signature that proves that the publisher created that message. Given that unique IDs are created for each channel (known as “roots”), only those parties who are authorized are able to read and reconstruct the entire message stream.

To approve a transaction that is sent to the Tangle via MAM, computational resources based on Proof of Work (PoW) algorithms are used to find the answer to a simple cryptographic puzzle. These algorithms and the underlying peer-to-peer protocols use low processing resources, making them well-designed for small devices (eg, sensors) [23]. PoW can either be done locally on the device itself or externally via an IOTA node or a special API.

To test the performance of sending IOTA transactions via MAM, we assessed the time required to both create and attach 300 anonymized FHIR records. The choice for 300 messages was based on the intent to compare the performance of our system with previous research [22].

The message was created on the local hardware, whereas attachment of the message was performed via an external API. To create the message locally, we used a dummy C# Xamarin mobile phone app [24] that uses the Tangle.Net.Mam Nuget to generate MAM transactions [25] together with our FHIR code [26], running on a Samsung Galaxy S8 phone with 2.3 GHz Quad-Core Exynos M2 Mongoose. For the attachment, we used the external PoW via an API to Powsrv, which uses the “PiDiver” hardware [27] and uses a random selection of healthy IOTA nodes on the IOTA Mainnet to take different server response times into account. The use of this external API speeds up the sending process and reduces the device power consumption [27].

#### Public IOTA Plus Private IPFS

For the second design of our system, we included a distributed file storage system (IPFS) to account for two key regulatory considerations: (1) uncertainty with regards to what anonymization techniques are legally sufficient to transform PHI into anonymized data and (2) to minimize both the reversal and linkability risks.

The IPFS is a peer-to-peer distributed file system based on content-addressed hyperlinks. As such, it takes files and manages them based on their content, storing them and tracking their version using a generalized Merkle directed acyclic graph. These Merkle trees, or hash trees, allow secure verification of the contents of large data structures, using cryptographic hash functions that map data of arbitrary sizes to data of a fixed size (hash).

Advantages of this technology include (1) data stored on IPFS are not automatically distributed between all participants and only shared in the case of a request, (2) IPFS nodes are able to delete specific data at any given point in case of a request, and (3) it is easy to prove whether an input will result in a given hash, but incredibly difficult to derive the input from a hash [28].

In this design, the aggregated JSON files containing multiple individual FHIR records were uploaded into a private IPFS cluster via a writable IPFS gateway. Every participant of this network was publicly known and can be held accountable in the case of noncompliance with a data deletion request from the data subject. Therefore, this private setup allowed for the specification of the number of backup copies in the network, and for the definition of automatic rules, such as when to delete data in the case of a patient request. A previous study provides more details on how to set up these specific rules on an IPFS cluster [29].

To link IPFS transactions to the authenticated and undeletable MAM transactions, hashes of the IPFS content were then shared via a MAM stream using the previously described IOTA libraries. For simplicity, we used the SHA256-256 hash function with Base58 encoding, which is the default hash function of IPFS.

The performance of this setup was assessed in two parts: (1) the time required to upload and generate a hash of the aggregated FHIR records using a writable IPFS gateway and (2) the time required to send this hash to the IOTA Tangle using MAM. This second test was also repeated 300 times using the same setup as in the previous design.

### Key Exchange Module

The key exchange module allows the establishment of a patient-provider communication channel for the FHIR records to be exchanged. In this key exchange process, two parties exchange cryptographic keys, allowing them to exchange encrypted messages exclusively. The design of this module is dependent on the data logging and storage layer; therefore, two variations were also used in this work.

#### Public IOTA

**Figure 5:**
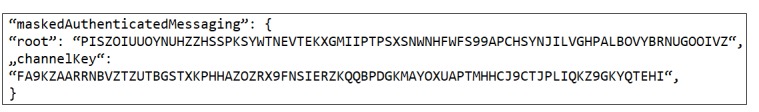
The Masked Authenticated Messaging (MAM) root and channel key of the in-person key exchange.

**Figure 6:**
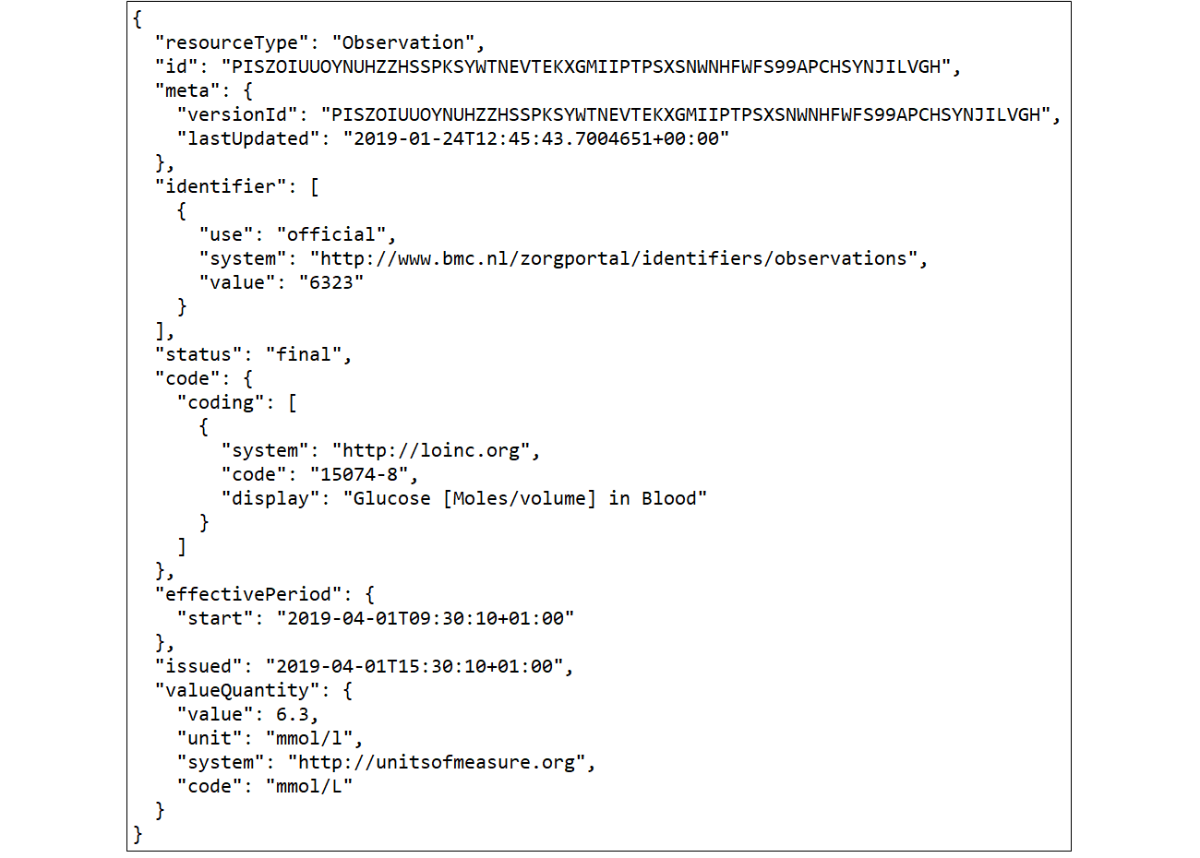
Anonymized JSON FHIR (JavaScript Object Notation Fast Healthcare Interoperability Resources) record stored on the public IOTA ledger with a pseudonymized ID consisting of the first 64 letters of the Masked Authenticated Messaging (MAM) root.

In this design, an initial exchange of private data in person or via a different non-DLT method was required (to maintain GDPR compliance). We assumed an initial in-person key exchange where, during the first visit to the physician’s office, the patient shared his or her channel keys using their mobile phone’s near field communication (NFC) chip (tapped on a receiver that the physician owns, linked to the EHR). The information included in this key exchange includes the MAM root and channel key (Figure 5). When channel keys are exchanged with a physician, the physician can then retrieve and authenticate the anonymized BG monitoring device data stream(s) that reside on the Tangle, in a similar JSON format to Figure 6. You may notice that the ID of the JSON is pseudonymized with the first 64 letters of the MAM root (Figure 5), which is used to help the physician to uniquely identify the patient.

**Figure 7:**
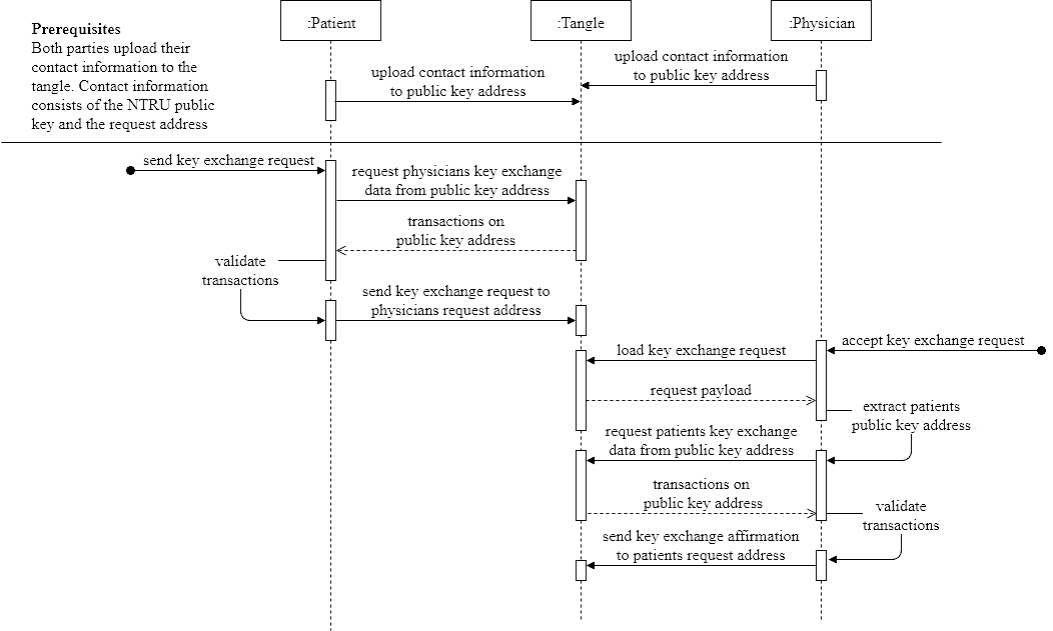
Steps for a remote key exchange via a public key exchange. NTRU: Nth degree‐truncatedpolynomial ring units.

At any point in time, patients can revoke access to their data stream by simply updating their MAM channel’s authorization key. If multiple doctors have access to the same channel, the parties who should continue to have access would get the new channel key through an additional key exchange process.

#### Public IOTA Plus Private IPFS

In this design, the initial exchange of keys and personal data can either take place in person during the first visit to the physician’s office or remotely via a public key exchange. Although the exchange of keys in person is the most secure option, there are use cases in which it is more convenient or not possible to exchange information unless it is done remotely (eg, follow-up remote consultation or providing data access to researchers). For this variation, we used a remote public key exchange system which assumes that both patient and physician have their contact information (an Nth degree‐truncated polynomial ring units [NTRU] public key uploaded to their public key IOTA address along with a link to their request address) published to the Tangle.

As shown in Figure 7, the steps for key exchange in this case are (1) the patient sends a key exchange request to the physician’s request address, (2) the physician decrypts this request with his NTRU private key (due to the nature of NTRU, only he can decrypt the data), (3) the physician confirms the key exchange request by sending his details to the patient’s request address, and (4) the channel access data can now be generated from the portions sent in steps 1 and 3, and both parties can now send encrypted data to the secured channel’s address.

## Results

We assessed (1) the performance of the data processing module, (2) the time required to send and store FHIR records in a DLT system by comparing the performance of the data storage and logging module for each of the two system designs, and (3) the time required for the remote key exchange process.

### Data Processing

For the public IOTA design, after the anonymization step, the size of the JSON file containing one BG FHIR record decreased from 2509 bytes to 857 bytes. During the aggregation step of the public IOTA plus private IPFS design*,* we combined the BG FHIR records for the equivalent of one-quarter of patient monitoring. This resulted in a JSON file with an approximate size of 34 MB. After this aggregation, and after also applying the JavaScript Obfuscator, the file size increased to 64 MB. Finally, after applying the AES256-GCM encryption, the resultant file had a size of 93 MB. It is important to note that the encryption time depended both on the encryption algorithm and processing hardware used. In this study, using the AES256-GCM algorithm and a Samsung Galaxy S8 device (2.3Ghz Quad-Core Exynos M2 Mongoose), it took less than 0.1 seconds to encrypt a 93 MB file which, in the larger picture of total transaction time, is negligible.

### Data Storage and Logging

#### Public IOTA

Table 1 shows the time required to both create and attach an anonymized FHIR record (with 857 bytes) with MAM. The total time required to create and attach a single message was mean 6.1 (SD 1.9) seconds (create: mean 3.5, SD 1.1 seconds; attach: mean 2.5, SD 0.8 seconds).

**Table 1:** Transaction times for storing and logging anonymized records of Fast Healthcare Interoperability Resources on the public IOTA design and public IOTA plus private InterPlanetary File System (IPFS) design and remote key exchange times.

Design and action			Trials	Time (ms), mean (SD)	Time (ms), range	Variance (ms^2^)
**Public IOTA**						
	Create		300	3525 (1182)	2042-8100	1,397,997
	Attach		300	2545 (765)	1357-8923	584,728
**Public IOTA + private IPFS**						
	Create		300	3636 (1371)	2249-12,673	18,794,477
	Attach		300	3522 (576)	2161-5146	331,554
	**Remote key exchange**					
		Send request	10	5160 (1801)	3100-8500	3,247,111
		Accept request	10	5790 (1253)	4000-8500	1,572,111

#### Public IOTA Plus Private IPFS

Next, we looked at the time required to upload and generate a hash of the aggregated FHIR record (93 MB) using a writable IPFS gateway. The hashing of this file was almost instantaneous; therefore, the total time required was fully dependent on the user’s bandwidth speed to upload this file. Our upload speed was on average 3 MB/s, which led to a total upload time of approximately 31.0 (SD 8.5) seconds. After uploading the FHIR records on the IPFS gateway, a hash was returned with a 46-character format similar to QmP543pymsKVHUdMg YQzSRbG7HoDSrVajhVRfrbtvhnGAQ. The mean times required to send this hash to the IOTA Tangle using MAM are presented in Table 1.

Using this public IOTA plus private IPFS design, the total amount of time required to share an aggregated quarter of glucose data was mean 38.1 (SD 13.4) seconds (upload: mean 31.0, SD 8.5 seconds; create: mean 3.6, SD 1.4 seconds; attach: mean 3.5, SD 0.6 seconds).

### Key Exchange

With the remote setup described in the public IOTA plus private IPFS design*,* we were able to exchange keys remotely and establish a secure connection in mean 10.9 (SD 3.1) seconds (send request: mean 5.2, SD 1.8 seconds; accept request: mean 5.8, SD 1.3 seconds) via the IOTA Tangle (Table 1).

## Discussion

### Principal Findings

Systems based on DLT hold great potential to help improve health information exchange. However, their design needs to comply with the latest regulations (eg, GDPR) to minimize the risk of inappropriate processing and/or storage. In this study, we assessed the potential of one specific DLT (IOTA) for the exchange of BG data, by designing and developing a proof-of-concept system considering important regulatory considerations. Such systems should also meet satisfactory performance and usability from the user perspective; therefore, we tested the performance of our design using a variety of measures.

Given the current open questions around data anonymization, an optimal system does not exist at this stage. Therefore, we designed two variations. Each of these have their own pros and cons, including (1) data reversal risk, (2) data linkability risks, (3) processing time, (4) file size compatibility, and (5) overall system complexity. This assessment is summarized in Table 2.

### Reversal Risk

Reversal risk represents the risk of being able to reconstitute the original data from anonymized or modified data. In the public IOTA design, given that FHIR records uploaded into the Tangle were stripped of any personal data, this risk is not applicable. For the public IOTA plus private IPFS design, the hash that is uploaded into the Tangle is the result of multiple processing layers (aggregation, obfuscation, and encryption). Reverse engineering this hash into the original file is, computationally, a massive task since it requires trialing the immense number of possible combinations of inputs, which can range from a few bytes to hundreds of terabytes in size. Even in the future, with the natural advancements in computing, it is difficult to imagine that it will be possible to extract the data from one 46-letter hash, which stands for 93 MB of obfuscated and encrypted data.

**Table 2:** Summary of the advantages and disadvantages of the two variations of the proposed proof-of-concept.

Feature	Public IOTA	Public IOTA + private IPFS^a^
Reversal risk	N/A^b^	Low
Linkability risk	Medium	Low
Processing time	Low	Low
File size compatibility	Small files	Any file size
Complexity	Low	Medium

^a^IPFS: InterPlanetary File System.

^b^N/A: not applicable.

### Linkability Risk

The linkability risk represents the risk that it is possible to link anonymized data to an individual by examining patterns of usage or context, or by comparison to other pieces of information. In the public IOTA design, by tracking the frequency of the uploads of data into the Tangle, plus reading the actual BG levels, it may be possible to link this information to a particular individual. A possible solution could entail the encryption of the raw BG levels. However, in the light of GDPR, any encryption algorithm is susceptible of being reversed in the future [9]; therefore, encrypted raw data can potentially be decrypted and the actual glucose levels shown. Given the cases of reidentification of anonymized health records reported in the literature [13], one should be careful not to assume that anonymized PHI can be uploaded to a public DLT without any risk. The exchange of multiple keys during the first appointment between patient and provider could be another solution to reduce the amount of PHI per MAM stream and therefore reduce the linkability risk in this design. This solution has not been explored in this work.

For the public IOTA plus private IPFS design, each resulting hash is unique, so there is no obvious way to cross-analyze the data and therefore determine who these data belong to.

### Data Processing Times

The message transaction times obtained were in line with previous research [22]. The overall low transaction times for messages on the IOTA DLT (on average between 6.1 to 38.1 seconds) suggest that both designs can be used for a continuous data stream. It is important to note here that, by definition, one MAM message actually consists of three transactions, which in theory could be performed in parallel. With the implementation of this code optimization (dividing the average times shown in Table 1 by 3), the actual average time per message creation and attachment would be 1175 ms and 848 ms, respectively. This would result in a total of approximately 2 seconds per IOTA message sent via MAM.

With hardware acceleration [27] of PoW, it is possible to achieve a PoW time of 300 ms. The overall response time of the external API was 848 ms, suggesting a further API bottleneck of approximately 548 ms. To achieve faster transaction times, users could set up their own hardware-accelerated IOTA nodes rather than using the centralized API service. Increasing the number of users running their own PoW also increases the overall health of the network, as it increases its decentralized character.

Specifically for the public IOTA plus private IPFS design, because the hashing itself is almost instant, the main limiting factor is the upload speed of the network. For this design, as a general recommendation, we recommend uploading of large files to the IPFS gateway only while connected to a fast WLAN network.

Finally, it is also relevant to note that for providers to read the BG data, taking into account the use case of quarterly consultations, the public IOTA design will benefit from using a “prefetching” of this quarterly data into the provider’s EHR. For the public IOTA plus private IPFS design, given the relatively low reading times (available on request), we do not envision the need for prefetching.

### File Size Compatibility

In the public IOTA design, the time required to create and send a message via MAM fully depends on the size of the file. Larger files may therefore not be suitable to be exchanged using this design. In the public IOTA plus private IPFS design, because the hashes are usually much smaller than the initial data and they can be used to identify the data itself, we can conclude that this design is well-suited for the exchange of larger files.

### Complexity

Regarding the overall design complexity, the public IOTA design is significantly simpler compared with the public IOTA plus private IPFS design. Two features, in particular, make the second design more complex: (1) the multiple steps involved in the data processing module require additional processing that can lead to larger file sizes (eg, using the quantum-secure NTRU encryption resulted in a file size of 821 MB) and (2) the setup of the private IPFS cluster requires additional implementation work.

The use case chosen in this paper focused on the remote exchange of BG measurements between a patient and a physician, where the physician “reads” the data on a periodic basis during the consultation, to review and update the diabetes management plan. Nevertheless, we are also confident that either design could be used in the use case of continuous streaming of individual BG measurements (ie, real-time data exchange and monitoring), as well in other types of remote health data exchange (such as the use cases proposed by Cohen et al [30]).

Related work in this field falls into two categories: (1) use of blockchain to improve health care data access and interoperability, and (2) assessment of how blockchain can be used considering data privacy regulations.

One of the first works describing the use of blockchain to tackle interoperability barriers was authored by the MedRec team [31]. In this work, a permissioned blockchain network based on a proof-of-work incentive was proposed to facilitate EHR data sharing and authentication. Following this work, Zhang et al [32] took a step further and described how EHR data could be securely and scalably shared to improve collaborative clinical decision support. Specifically, an FHIR-based smart contract system was proposed for exchanging health data, in which the blockchain stores encrypted metadata and an off-chain solution is used for clinical data.

This work does present an FHIR-based provider-provider solution, although our work proposes a complementary FHIR-based solution for patient-provider data exchange. On the topic of FHIR-based interoperability, Peterson et al [33] described a blockchain solution using a new type of consensus mechanism: an FHIR-based “Proof of Interoperability.” This approach is unique in the sense that it takes security and interoperability as the central tenet of its core design.

In contrast to the previous studies, it is important to note two additional articles positioning blockchain as a tool for patient-provider data exchange instead of provider-provider exchange. Ichikawa et al [34] developed a mHealth system using a mobile phone app that enables a patient-provider exchange of information but for the particular goal of insomnia cognitive behavioral therapy. Balsari et al [35] proposed a use case leveraging the high mobile phone penetration and availability of unique ID systems in India to facilitate health data exchange between more than 500 million Indian citizens and their providers.

The use of blockchain in light of data privacy regulations has been previously described by Zyskind et al [36] across three domains: data ownership, data transparency and auditability, and access control. This article describes a method similar to our second proposed design, namely an on-chain solution combined with off-blockchain storage to construct a personal data management platform focused on privacy. Conversely, Al Omar et al [37], arguing that decentralized approaches for data exchange may fail to ensure overall privacy, applied additional cryptographic functions and data processing procedures to encrypt patients’ data and to ensure pseudonymity.

### Limitations

In this work, we address some of the points of tension between GDPR and DLT by proposing two designs that enable the exchange of PHI using this technology. However, future work needs to address in more detail the remaining points of conflict, such as how to identify a data controller in a public DLT ecosystem formed by multiple health care stakeholders or how to collect and manage an individual’s express consent for the processing of health data.

It is also important to note a few limitations with the designs proposed in this study. First, using a patient’s mobile phone’s NFC chip for key exchange is not currently practical because it requires every physician’s office to have an NFC reader connected to the facility’s EHR. Alternatives to this could be an in-person key exchange method or secure local wireless network. Second, in our testing, the continuous glucose monitoring device generated a data point every 10 minutes, meaning that significant battery usage could be expected over the day to create all FHIR records. The actual implications on the device battery may constitute an important limitation requiring further investigation.

Finally, it is also important to point out that the experiments run in this study were carried out on a Samsung Galaxy S8 only. The CPU of this device was used to calculate the resource hashes locally; therefore, it can be expected that different mobile devices will require different times to create and attach an FHIR resource.

### Conclusion

The design of a DLT-based system for health data exchange needs to take into careful consideration the respective use case. In this paper, we proposed and developed two possible designs that aim to be compliant with recent data privacy regulations, minimizing any risks of misappropriate data processing and returning satisfactory performance and usability. One design was based solely on the public distributed ledger IOTA and the second used IOTA plus a private IPFS cluster. Our findings suggest that the first design is simpler to implement but requires special attention to minimize the risk of personal data linkability, and that the second design allows for the exchange of larger files at the expense of higher complexity.

## Abbreviations

API: application programming interface
BG: blood glucose
DLT: distributed ledger technology
EHR: electronic health record
FHIR: Fast Healthcare Interoperability Resources
GDPR: General Data Protection Regulation
IPFS: InterPlanetary File System
JSON: JavaScript Object Notation
MAM: Masked Authenticated Messaging
NFC: near field communication
NTRU: Nth degree‐truncated polynomial ring units
PHI: protected health information
PoW: Proof of Work

## References

[1] Health Information Technology Policy Committee (2015). Report to Congress: Challenges and Barriers to Interoperability.

[2] Office of the National Coordinator for Health Information Technology (ONC) (2015). Report to Congress: Report on Health Information Blocking.

[3] Dean AA (2018). Journal of AHIMA.

[4] Mello MM, Adler-Milstein J, Ding KL, Savage L (2018). Legal barriers to the growth of health information exchange-boulders or pebbles?. Milbank Q.

[5] Gordon WJ, Wright A, Landman A (2017). NEMJ Catalyst.

[6] Gordon WJ, Catalini C (2018). Blockchain technology for healthcare: facilitating the transition to patient-driven interoperability. Comput Struct Biotechnol J.

[7] Kuo TT, Kim HE, Ohno-Machado L (2017). Blockchain distributed ledger technologies for biomedical and health care applications. J Am Med Inform Assoc.

[8] Angraal S, Krumholz HM, Schulz WL (2017). Blockchain technology: applications in health care. Circ Cardiovasc Qual Outcomes.

[9] European Union Blockchain Observatory and Forum (2019). Blockchain and the GDPR.

[10] (2016). Official Journal of the European Union.

[11] Working Party on the Protection of Individuals with Regard to the Processing of Personal Data (2014). Opinion 05/2014 on Anonymisation Techniques.

[12] Ohm P (2010). Broken promises of privacy: responding to the surprising failure of anonymization. UCLA Law Rev.

[13] El Emam K, Jonker E, Arbuckle L, Malin B (2015). Correction: a systematic review of re-identification attacks on health data. PLoS One.

[14] McCallister E, Grance T, Scarfone K (2010). Guide to Protecting the Confidentiality of Personally Identifiable Information (PII).

[15] Golden SH, Sapir T (2012). Methods for insulin delivery and glucose monitoring in diabetes: summary of a comparative effectiveness review. J Manag Care Pharm.

[16] Health Level Seven International (HL7).

[17] Kawamoto K, Hongsermeier T, Wright A, Lewis J, Bell DS, Middleton B (2013). Key principles for a national clinical decision support knowledge sharing framework: synthesis of insights from leading subject matter experts. J Am Med Inform Assoc.

[18] Mandel JC, Kreda DA, Mandl KD, Kohane IS, Ramoni RB (2016). SMART on FHIR: a standards-based, interoperable apps platform for electronic health records. J Am Med Inform Assoc.

[19] (2014). Bluetooth SIG.

[20] Kachalov T (2018). Github.

[21] Nechvatal J, Barker E, Bassham L, Burr W, Dworkin M, Foti J, Roback E (2001). Report on the Development of the Advanced Encryption Standard (AES). J Res Natl Inst Stand Technol.

[22] Brogan J, Baskaran I, Ramachandran N (2018). Authenticating health activity data using distributed ledger technologies. Comput Struct Biotechnol J.

[23] Popov S (2018). The Tangle.

[24] Pact Care BV (2018). Github.

[25] Felandil S Nuget.

[26] Pact Care BV (2018). Github.

[27] Pototschnig T (2018). Microengineer.

[28] Benet J (2019). IPFS - Content Addressed, Versioned, P2P File System (DRAFT 3).

[29] Protocol Labs (2019). IPFS.

[30] Cohen DJ, Keller SR, Hayes GR, Dorr DA, Ash JS, Sittig DF (2016). Integrating patient-generated health data into clinical care settings or clinical decision-making: lessons learned from Project HealthDesign. JMIR Hum Factors.

[31] Azaria A, Ekblaw A, Vieira T, Lippman A (2016). MedRec: using blockchain for medical data access and permission management. Proceedings of the 2nd International Conference on Open and Big Data.

[32] Zhang P, White J, Schmidt DC, Lenz G, Rosenbloom ST (2018). FHIRChain: applying blockchain to securely and scalably share clinical data. Comput Struct Biotechnol J.

[33] Peterson K, Deeduvanu R, Kanjamala P, Boles K (2016). ONC/NIST.

[34] Ichikawa D, Kashiyama M, Ueno T (2017). Tamper-resistant mobile health using blockchain technology. JMIR Mhealth Uhealth.

[35] Balsari S, Fortenko A, Blaya JA, Gropper A, Jayaram M, Matthan R, Sahasranam R, Shankar M, Sarbadhikari SN, Bierer BE, Mandl KD, Mehendale S, Khanna T (2018). Reimagining health data exchange: an application programming interface-enabled roadmap for India. J Med Internet Res.

[36] Zyskind G, Nathan O, Pentland A (2015). Decentralizing privacy: using blockchain to protect personal data. IEEE Secur Priv Work.

[37] Al Omar A, Bhuiyan MZ, Basu A, Kiyomoto S, Rahman MS (2019). Privacy-friendly platform for healthcare data in cloud based on blockchain environment. Future Gener Comp Sy.

